# Fresh Produce Safety and Quality: Chlorine Dioxide’s Role

**DOI:** 10.3389/fpls.2021.775629

**Published:** 2022-01-11

**Authors:** Siva Kumar Malka, Me-Hea Park

**Affiliations:** Postharvest Research Division, National Institute of Horticultural and Herbal Science, Wanju-gun, South Korea

**Keywords:** chlorine dioxide, fresh produce, microbial safety, log reduction, produce quality, storage, shelf life, antimicrobial mechanism

## Abstract

Maintaining microbial safety and quality of fresh fruits and vegetables are a global concern. Harmful microbes can contaminate fresh produce at any stage from farm to fork. Microbial contamination can affect the quality and shelf-life of fresh produce, and the consumption of contaminated food can cause foodborne illnesses. Additionally, there has been an increased emphasis on the freshness and appearance of fresh produce by modern consumers. Hence, disinfection methods that not only reduce microbial load but also preserve the quality of fresh produce are required. Chlorine dioxide (ClO_2_) has emerged as a better alternative to chlorine-based disinfectants. In this review, we discuss the efficacy of gaseous and aqueous ClO_2_ in inhibiting microbial growth immediately after treatment (short-term effect) versus regulating microbial growth during storage of fresh produce (long-term effect). We further elaborate upon the effects of ClO_2_ application on retaining or enhancing the quality of fresh produce and discuss the current understanding of the mode of action of ClO_2_ against microbes affecting fresh produce.

## Introduction

Fresh produce, including fruits and vegetables, is a good source of nutrients and an important component of a healthy and balanced diet. However, fresh produce is susceptible to microbial contamination, which may occur at any step of the food supply chain, from sowing the crop to delivering it to the customer. Furthermore, cross-contamination can occur during processing, packaging, or transporting fresh produce. Most common sources of food contamination are soil, animal manure, and irrigation water, and consuming contaminated food may lead to the outbreak of foodborne illnesses. In the United States of America, 340 foodborne outbreaks, from 2009 to 2018, were associated with fresh produce (CDC, 2018). Furthermore, 5,175 foodborne outbreaks were reported in Europe in 2019, involving 49,463 cases, 3,859 hospitalizations, and 60 fatalities (European Food Safety Authority, 2021). Foodborne diseases not only affect human health but also pose challenges to tourism, agricultural, and food industries, thereby seriously affecting socioeconomic development (WHO, 2015).

Microbial contamination can affect the quality and shelf-life of fresh produce. In 2010, an estimated 31% of the total food produce worth $161.6 billion was declared unfit for human consumption at the retail and consumer levels (Buzby et al., 2014). With an increased awareness of the importance of fresh produce consumption for a healthy lifestyle, the concerns of modern consumers regarding the freshness, appearance, and microbial safety of fresh produce have increased. At the retail level, primarily in supermarkets and hypermarkets, 15–30% of the fresh produce was rejected by consumers because of quality standards that over-emphasize appearance (FAO, 2011). Hence, microbial safety and high quality have emerged as a concern for the food industry and consumers.

Several physical and chemical disinfection methods have been used to reduce the microbial load on fresh produce (Deng et al., 2020; Chacha et al., 2021). However, a potent disinfection method must fulfill the following criteria: high efficacy against pathogens, ability to reduce microbial spoilage, potential to retain nutritional quality, no formation of intolerable levels of human toxic by-products or residues, and no environmental impact (Joshi et al., 2013). The efficacies of various physical disinfection methods, including high hydrostatic pressure, cold plasma, ultraviolet, ultrasound, pulsed, and ionizing radiation, have been examined. However, these methods have various disadvantages. For instance, ultrasound shows limited antimicrobial effect, ultraviolet light has low penetration and shade effect from complex surface properties of produce affects its efficiency, and pulsed light increases temperature that deteriorates the quality of treated produce (Deng et al., 2020).

Chlorine is the most commonly used chemical disinfectant in the food industry, which is effective against a broad range of pathogens and whose efficacy has been evaluated in a wide variety of fresh produce (Praeger et al., 2018). However, chlorine may react with natural organic matter and form halogenated by-products, such as trihalomethanes or haloacetic acids (Praeger et al., 2018). These by-products are carcinogenic and not environment-friendly. Moreover, owing to safety and efficacy concerns, the use of chlorine for the sterilization of fresh-cut produce has been banned in countries such as Belgium, Switzerland, and Netherlands (Deng et al., 2020). Therefore, several chemical alternatives, such as chlorine dioxide (ClO_2_), ozone, electrolyzed water, essential oils, high-pressure carbon dioxide, and organic acids, have been identified or proposed (Deng et al., 2020). For instance, electrolyzed water, and ozone are potent disinfectants; however, for the effective microbial reduction high concentration or prolonged exposure is required. Excessive usage of these treatments can negatively affect produce quality (Deng et al., 2020). Organic acids are safe and easy to use but their antimicrobial efficiency is limited. Essential oils are natural antimicrobial agents; however, it is practically difficult use these oils because of their hydrophobic, volatile and unstable nature (Deng et al., 2020).

Application of ClO_2_, an oxidative gas, is effective in controlling the bacterial, fungal, and viral contamination of fresh produce (Praeger et al., 2018; Sun et al., 2019). In contrast to chlorine, ClO_2_ neither produces toxic by-products nor does it alter the nutritive and organoleptic qualities of food products, and is effective over a wide pH range (pH 3–8). In addition, it is widely used as a bleaching agent in paper industry and as a disinfectant in laboratories, hospitals, public places, and other areas (Praeger et al., 2018). Owing to its efficacy and safety, ClO_2_ has been approved for the disinfection of fresh produce and in food processing industries (FDA, 2008). Recently, Praeger et al. (2018) and Sun et al. (2019) comprehensively reviewed antimicrobial activity of aqueous and gaseous ClO_2_, respectively. This review focuses on the effects of ClO_2_ application on the initial reduction in microbial growth (short-term effect) and the final reduction in microbial populations during the storage of fresh produce (long-term effect). We further discuss the efficacy of ClO_2_ application in maintaining the quality of fresh produce and the action mechanism of ClO_2_ against microbes affecting fresh produce.

## Modes of Chlorine Dioxide Application

Chlorine dioxide is a yellowish-green gas and is highly water soluble, approximately 10 times more soluble in water than chlorine, particularly in cold water. Moreover, it remains in solution as a dissolved gas without hydrolyzing. Hence, it can be used in aqueous as well as gaseous forms. The advantages and limitations of using aqueous and gaseous ClO_2_ are summarized in Table 1.

**TABLE 1:** Chlorine dioxide (ClO_2_) application in aqueous and gaseous form: advantages and disadvantages.

Aqueous application	Gaseous application
**Advantages** (Praeger et al., 2018)	**Advantages** (Sun et al., 2019)
Easy to handle, inexpensive	Higher antimicrobial activity
It can be used in the form of spray, immerse or washing	It can be applied as batch treatment or continuous treatment
Concentration and contact can be maintained	High accessibility to microbes irrespective of surface barriers
Easy to adopt in industrial washing lines	No water rinsing required after the treatment
	It can impact microbial internalization
	No issue of cross-contamination of wash water
**Disadvantages** (Praeger et al., 2018)	**Disadvantages** (Sun et al., 2019)
Produce surface properties can affect ClO_2_ accessibility to microbes	Needs onsite generation
Cross-contamination of wash water	Needs technical knowledge
Water rinsing is required after the treatment	laborious to perform, expensive
Residual moisture after the water rinsing can promote microbial growth	Explosive at higher concentration
Not suitable for dried foods	Challenging to maintain concentration and contact time
Relatively less effect on microbial internalization	Difficult to implement at industry scale

Aqueous ClO_2_ solution can be used to spray, immerse, or wash fresh produce, as it ensures adequate ClO_2_ concentration and contact time, both of which are the determinants of its efficacy against pathogens. Moreover, aqueous ClO_2_ application is relatively easy to implement or adopt in the existing washing lines in food industries without modifying subsequent processes (Wu and Kim, 2007). However, water rinsing, an additional step, is required following aqueous ClO_2_ treatment, resulting in residual moisture on the produce surface that may stimulate microbial growth (Trinetta et al., 2011).

In contrast to aqueous ClO_2_, gaseous ClO_2_ is more effective against pathogens because of its higher potential to reach microbes irrespective of the surface irregularities of fresh produce (Han et al., 2001a). ClO_2_ is generally produced by the reaction of an acid with sodium chlorate or sodium chlorite and chlorine gas (Praeger et al., 2018). As gaseous ClO_2_ application does not require water, the risk of cross-contamination with recycled wash-water can be avoided. However, the major limitation of gaseous ClO_2_ application is its on-site production, as it cannot be compressed and stored or transported under pressure (EPA, 1999). Moreover, ClO_2_ production is laborious and expensive, and it is technically challenging to maintain a precise ClO_2_ concentration during gaseous treatment (Wu and Kim, 2007).

Alternatively, several packaging systems that can generate and release ClO_2_ have been developed. In these systems, materials that generate gaseous ClO_2_, including perforated sachets, pouches, tablets, films, and pads, are incorporated into the packaging system using different methods (Singh et al., 2021). Furthermore, for the development of an active packing material, factors, such as the release rate of the active material, its efficacy against microbes, and the maintenance of shelf-life of the product to be packed, are taken into consideration. These packaging systems are often designed to be used in combination with other technologies, such as modified atmosphere packaging (MAP).

## Modes of Chlorine Dioxide Action

### Antimicrobial Mechanisms

**FIGURE 1:**
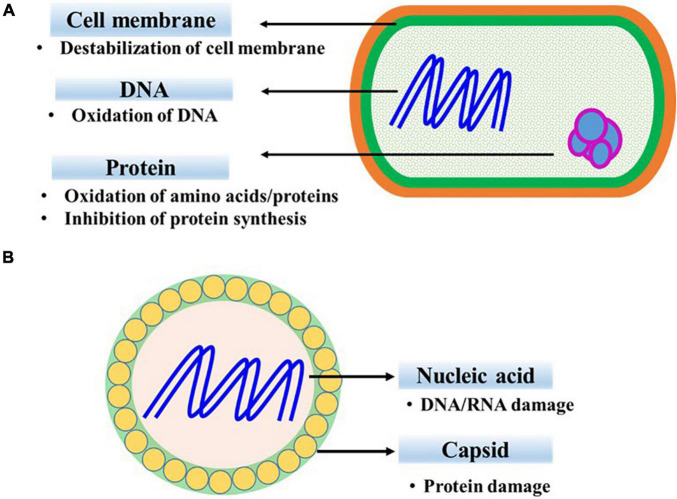
Mechanisms of chlorine dioxide against bacteria **(A)** and viruses **(B)**.

The antibacterial mechanism of ClO_2_ includes destabilization of the cell membrane, alteration of membrane permeability, and interruption of protein synthesis (Figure 1A). ClO_2_ reacts with oxygenated compounds and proteins in cell membranes, resulting in the disruption of cell metabolism (Praeger et al., 2018). Membrane damage in ClO_2_-exposed *Bacillus subtilis* spores inhibits their development after germination (Young and Setlow, 2003). Moreover, ClO_2_ oxidizes the exposed sulfhydryl groups of cell surface proteins, thereby causing membrane damage and increasing outer membrane permeability. Loss of permeability control, evident from the efflux of K^+^ ions, results in the destruction of transmembrane ionic gradient in *Escherichia coli* (Berg et al., 1986). Furthermore, loss of cell activity or cell death in ClO_2_-treated *Pseudomonas aeruginosa* and *Staphylococcus aureus* is correlated with the increased permeability of inner and outer cell membranes and the subsequent release of vital nuclear materials (Ofori et al., 2018). At higher concentrations, ClO_2_ induces accumulation of malondialdehyde (MDA) content, indicating the occurrence of membrane peroxidation (Bridges et al., 2020). However, previous studies based on transmission electron microscopy did not reveal significant morphological damage or cell lysis (Ofori et al., 2018; Bridges et al., 2020). Additionally, amino acids, including cysteine, tyrosine, tryptophan, histidine, and proline, are responsive to ClO_2_, with their order of reactivity from high to low, respectively (Sharma and Sohn, 2012).

The virucidal mechanism of ClO_2_ varies depending on the composition and three-dimensional structure of viral proteins and nucleic acids (Figure 1B). Degradation of viral capsid proteins inhibits the attachment of ClO_2_-exposed bacteriophages to host cells (Ge et al., 2021). Similarly, the ClO_2_-mediated destruction of glycoproteins affects viral attachment to cell receptors and alters the life cycle of porcine reproductive and respiratory syndrome virus (Zhu et al., 2019). In addition, the denaturation of viral proteins has been reported to be involved in the inactivation of human rotavirus (Xue et al., 2013). ClO_2_ damages the 5’ non-coding region in the viral genome that is necessary for formation of new virus particles within the host cells (Li et al., 2004; Jin et al., 2013). Furthermore, RNA damage, in addition to protein damage, has been attributed to the inactivation of poliovirus (Simonet and Gantzer, 2006).

The fungicidal mechanism of ClO_2_ involves disruption of both the plasma and mitochondrial membranes (Zhang and Fu, 2018; Lin et al., 2021). ClO_2_ treatment causes ion leakage, inhibition of key enzyme activities in metabolic pathways, and alteration of cell structure in *Saccharomyces cerevisiae* (Zhu et al., 2013). Further, ClO_2_ induces membrane lipid peroxidation, which is evident by enhanced MDA levels in *Penicillum expansum* (Zhang and Fu, 2018).

### Potential Mechanisms Regulating Fresh Produce Quality

**FIGURE 2:**
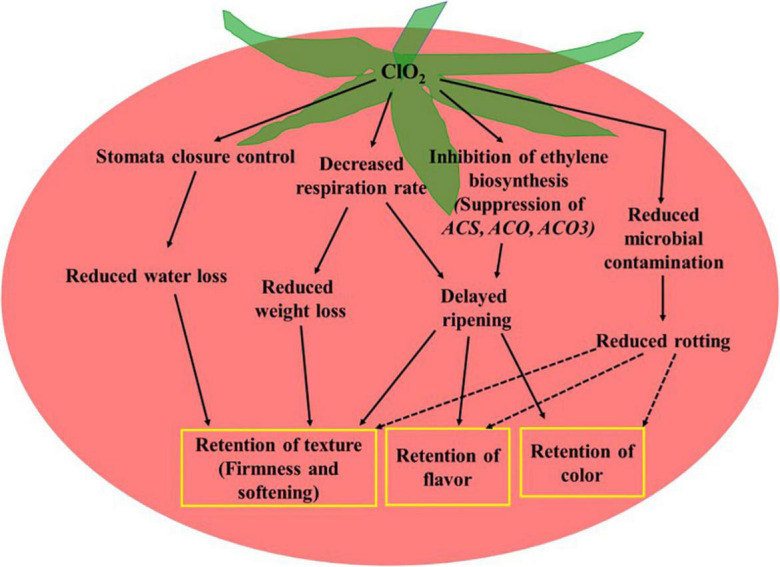
Potential mechanisms of chlorine dioxide regulating fresh produce quality. Dashed arrows indicate indirect effect.

The mechanisms underlying the regulation of fresh produce quality by ClO_2_ include its impact on respiration rate and ethylene biosynthesis (Figure 2). ClO_2_-mediated inhibition of ethylene biosynthesis, brought about by the suppression of ethylene biosynthesis-related genes, including *ACS2*, *ACO1*, and *ACO3* (Guo et al., 2013, 2014), alters the physiological and biochemical changes that occur during fruit maturation and senescence. Reduced respiration rate and transpiration delay the consumption of nutrients and water, which directly influences fruit firmness, mass loss, and softening (Chen and Zhu, 2011; Guo et al., 2013, 2014). The quality of fresh produce during storage depends on the correlation between cellular energy and redox status. For example, delayed senescence in ClO_2_-treated longan fruit has been reported to be associated with an altered redox state and increased cellular energy (Chumyam et al., 2016). Moreover, reduced microbial incidence in ClO_2_ treated produce leads to quality retention and shelf-life extension (Islam et al., 2017).

## Effects of Chlorine Dioxide Treatment on Microbes Affecting Fresh Produce

Chlorine dioxide concentration and contact time are crucial in determining the efficacy of ClO_2_, which may also vary with the type of microorganism and fresh produce. The short-term and long-term efficacy of ClO_2_ in inhibiting the growth of preexisting or artificially inoculated microorganisms have been demonstrated in a wide variety of fresh produce (Tables 2, 3). Furthermore, some reports suggested that artificially inoculated human pathogens, such as *E. coli*, *Salmonella* spp., and *Listeria monocytogenes*, exhibit higher inactivation on fresh produce than natural microflora after ClO_2_ application (Praeger et al., 2018).

**TABLE 2:** Effects of chlorine dioxide on short- and long-term reduction of microorganisms in vegetables.

Produce	Microorganism	Treatment conditions			Log reductions		Storage conditions	References
		Mode	Concentration	Duration	Short-term	Long-term		
Lettuce	Total aerobic bacteria	Aq	50 ppm	10 min	1.77	0.9	8 days, 4°C	Kim et al., 2007
	Yeasts and molds				1.34	1.16		
	Coliforms				1.1	0.9		
Lettuce	*Escherichia coli* O157:H7	Aq	20 ppm	10 min	1.44	1.38	4 days, 4°C	Kim et al., 2008
	*Salmonella*				1.95	1.91		
	*Listeria monocytogenes*				1.2	0.99		
Lettuce	*Escherichia coli* O157:H7, L. monocytogenes	Aq	3, 5 ppm	5 min	5.6	Unchanged	9 days, 4°C	Rodgers et al., 2004
Lettuce	*Escherichia coli O157:H7*	G	5 ppm	10 min	5	NA	7 days, 4°C	Mahmoud and Linton, 2008
	*Salmonella enterica*				5	NA		
	Mesophilic				NA	2.7		
	Psychrotrophic				NA	2		
	Yeast and molds				NA	2.2		
Spinach	*Escherichia coli O157:H7*	Aq	100 ppm	5 min	2.6	0.13	7 days, 7°C	Lee and Baek, 2008
Tomato	*Salmonella*	Aq	10 ppm	5 min	2.53	1.61	10 days, 4°C	Song et al., 2011
	*Escherichia coli* O157:H7				2.26	2.23		
Tomato	*Alternaria alternata*	G	10 ppm	1 min	2.71	Completely inactivated	10 days, 25°C	Trinetta et al., 2013
	*Stemphylium vesicarium*				2.63	Completely inactivated		
Tomato	*Salmonella enterica*	G	8 ppm	60 s	2.94	NA	28 days, 25°C	Trinetta et al., 2010
			10 ppm	120 s	3.86	NA		
			10 ppm	180 s	4.87	NA		
	Yeast and molds					1.57, 1.47, 1.54		
	*Mesophilic bacteria*					1.16, 2.81, 3.17		
Tomato	*Listeria monocytogenes, Salmonella*	G	0.5 ppm	12 min	>5	NA	28 days, 22°C	Bhagat et al., 2010
	Mesophilic				NA	0.6		
	Yeast and molds				NA	0.1		
Tomato	*Escherichia coli*	G	3.5 ppm	14 days	2.9–4.7	3.08	14 days, 20°C	Sun et al., 2017b
	*Alternaria alternata*				1.6–4.0	2.85		

*Aq, aqueous; G, gaseous; NA, not available.*

**TABLE 3:** Effects of chlorine dioxide on short- and long-term reduction of microorganisms in fruits.

Produce	Microorganism	Treatment conditions			Log reduction		Storage conditions	References
		Mode	Concentration	Duration	Short-term	Long- term		
Apples	*Escherichia coli O157:H7*	Aq	3, 5 ppm	5 min	5.6	Unchanged	9 days, 4°C	Rodgers et al., 2004
	*Listeria monocytogenes*				5.6	Unchanged		
Apples	*Salmonella*	G	4.1 ppm	6–25 min	4.21	NA	10 days, 10°C	Sy et al., 2005b
	Yeasts and molds				1.68	NA		
Apple	*Alicyclobacillus acidoterrestris*	G	0.39 ppm	1 h	2.7	NA	7 days, 4°C	Lee et al., 2006
			0.50 ppm	2 h	3.7			
			0.60 ppm	3 h	4.5			
Blueberry	*Salmonella enterica*	G	1.5 ppm	NA	4.45	5.63	NA	Annous et al., 2020
			3 ppm		5.63			
Blueberry	*Listeria monocytogenes*	Aq	1, 3, 5, 10, 15 ppm	10 s, 1, 5, 10, 20, 30 min; 1, 2 h	0.07–4.88	NA	NA	Wu and Kim, 2007
	*Pseudomonas aeruginos*				0.15–4.48			
	*Salmonella*				0.12–3.32			
	*Staphylococcus aureus*,				0.21–4.56			
	*Yersinia enterocolitica*				0.18–3.69			
Blueberries	Total aerobic bacteria	Aq	100 ppm	10 min	1.4–1.5	1.46 (20°), 1.14 (4°C)	12 days, 20 or 4°C	Chun et al., 2013
	Yeasts and molds				0.8–0.9	1.61 (20°), 0.35 (4°C)		
Strawberry	*Escherichia coli O157:H7*	G	0.5, 1, 1.5, 3, 5 ppm	10 min	4.6	NA	16 days, 22°C	Mahmoud et al., 2007
	*Listeria monocytogenes*				4.7	NA		
	*Salmonella*				4.3	NA		
	Mesophilic bacteria				NA	3		
	Psychrotrophic bacteria					1.7		
	Yeast and mold					1.9		
Strawberry	*Escherichia coli* O157:H7	Aq	5 ppm	5 min	5.6 l	Unchanged	9 days, 4°C	Rodgers et al., 2004
	*Listeria monocytogenes*				5.6 l	Unchanged		
	Psychrotrophic				2.5	2.5		
	Lactic acid bacteria				1.5	1.7		
	Yeast and mold				1.1	1.1		
Mulberry	Mesophilic,	Aq	20, 60, 80 ppm	5, 10, 15 min	2.4–2.8	2.0–2.6	14 days, -1°C	Chen et al., 2011
	Psychrotrophic				2.4–2.5	2.3–2.5		
	Lactic acid bacteria				1.4–1.5	1.5–1.7		
	Yeast and mold				1.0–1.1	0.9–1.1		
Cantaloupe	*Escherichia coli O157:H7*,	G	5.0 ppm	5.5 min;	5	NA	12 days, 22°C	Mahmoud et al., 2008
	*Listeria monocytogenes, Salmonella;*				5	NA		
	Mesophilic		0.5, 1, 1.5, 3, 5 ppm	0, 2, 4, 6, 8, 10 min	NA	2.4		
	Psychrotrophic bacteria,					4.1		
	Yeasts and molds					2.2		
Cantaloupe	*Escherichia coli* O157:H7, *L. monocytogenes*	Aq	3, 5 ppm	5 min	5.6	Unchanged	9 days, 4°C	Rodgers et al., 2004
Grape fruit	*Escherichia coli*	G	5 ppm	24 h	Non-detectable	NA	42 days, 10°C + 7 days, 20°C	Sun et al., 2017a
	*Penicillium digitatum*		60 ppm		Non-detectable	NA		
	*Xanthomonas citri*		14.5, 29 ppm		Non-detectable	NA		
	Total aerobic bacteria,				NA	0.95		
	Yeast and mold				NA	0.94		
Peaches	*Salmonella*,	G	4.1 ppm	6–25 min	3.23	NA	10 days, 10°C	Sy et al., 2005b
	Yeasts and molds				2.68	NA		

*Aq, aqueous; G, gaseous; NA, not available.*

### Short-Term Effects

#### Vegetables

Washing leafy vegetables with aqueous ClO_2_ was effective in inactivating natural microflora. For instance, the initial populations of aerobic mesophilic, aerobic psychrotrophic, and lactic acid bacteria, yeast, and molds, in raw asparagus lettuce slices decreased by 1–3 log upon treatment with 100 ppm ClO_2_ for 20 min (Chen et al., 2010). Furthermore, exposure to 3 ppm ClO_2_ for 1 min reduced epiphytic microbiota on fresh-cut iceberg lettuce by 1–2 log (López-Gálvez et al., 2010). The efficacy of ClO_2_ against artificially inoculated pathogens, such as *E. coli*, *Salmonella* spp., and *L. monocytogenes*, has been extensively investigated in lettuce. A 2 min-long treatment with 100 or 200 ppm aqueous ClO_2_ in iceberg lettuce resulted in >1 log reduction in *E. coli* O157:H7 load (Keskinen et al., 2009). Similar results were obtained for *S. typhimurium* and *L. monocytogenes* inoculated on iceberg lettuce with lower ClO_2_ concentrations but longer exposure (10 min) (Kim et al., 2008). Moreover, Rodgers et al. (2004) observed >5 log reduction in the loads of *E. coli* O157:H7 and *L. monocytogenes* after ClO_2_ application (5 ppm for 5 min) on green leaf lettuce. Similar observations were made in whole heads of iceberg lettuce exposed to 5 ppm gaseous ClO_2_ for 15–20 min (Mahmoud and Linton, 2008). In spinach leaves, treatment with high ClO_2_ concentration for a short contact time (100 ppm for 5 min) or low ClO_2_ concentration with a long exposure time (10 ppm for 20 min) yielded approximately similar levels of pathogen reduction (Lee and Baek, 2008; Park and Kang, 2015b).

Several studies have investigated the disinfection of tomatoes using ClO_2_ (Praeger et al., 2018; Sun et al., 2019). For artificially inoculated human pathogens, 5–7 log reduction in microbial load has been observed with gaseous ClO_2_ concentrations < 1 ppm (Bhagat et al., 2010; Olanya et al., 2015; Netramai et al., 2016). For instance, a 12 min exposure to 0.5 ppm ClO_2_ resulted in >5 log reduction in *Salmonella* and *L. monocytogenes* loads in hydroponically grown tomatoes (Bhagat et al., 2010). With an increased exposure time (approximately 1 h), grape tomatoes exhibited >7 log reduction in the load of *Salmonella* spp., at 25°C (Netramai et al., 2016). Trinetta et al. (2010) evaluated the efficacy of short-term exposure of high ClO_2_ concentrations in the inactivation of *S. enterica* inoculated on tomatoes and observed that the initial populations (6 log) were reduced to 3 log, 2 log, and 1 log in response to 8 ppm ClO_2_ for 60 s, 10 ppm ClO_2_ for 120 s, and 10 ppm for 180 s, respectively. Previous studies suggest that the disinfection efficiency of ClO_2_ on tomatoes freshly spot-inoculated with *Salmonella* and *Erwinia carotovora* is higher than that on produce with desiccated inoculum (Pao et al., 2007). Moreover, tomato packaging with ClO_2_-generating materials, such as films, sachets, and pouches, is effective in achieving microbial reduction from 4 to 6 log to undetectable levels (Mahovic et al., 2007; Ray et al., 2013; Sun et al., 2017b; Zhou et al., 2018). Additionally, Trinetta et al. (2013) reported complete inhibition of the mycelial growth of *Alternaria alternate* and *Stemphylium vesicarium* using a 3 min-long ClO_2_ treatment. ClO_2_ efficiency has also been reported to increase with an increase in relative humidity and temperature (Park and Kang, 2015b,^2018^).

Antimicrobial efficiency of ClO_2_ has also been evaluated in other fresh vegetables. After ClO_2_ treatment, minimally processed carrots exhibited significantly decreased levels of mesophilic aerobic bacteria (1.9 log), psychrotrophs (1.7 log), lactic acid bacteria (2.6 log), and yeast (0.7 log) (Gomez-Lopez et al., 2007). Potato exposed to ClO_2_ for 5 h exhibited a 5 log and 6 log reduction in natural microflora and *Pseudomonas aeruginosa*, respectively (Wu and Rioux, 2010). ClO_2_ application for 30 min reduced the loads of *E. coli* O157:H7 or *L. monocytogenes* inoculated on surface-injured green peppers by 6.5 and 3.5 log, respectively (Han et al., 2000, 2001b). Similarly, ClO_2_ treatment effectively inactivated natural microbiota and inoculated *Salmonella* on the surface of chili peppers (Lee et al., 2018). Furthermore, disinfection of red chili pepper with ClO_2_ after hot-air drying significantly decreased *Bacillus cereus* spore populations below the detection limit (1.7 log) (Kim et al., 2017).

#### Fruits

Aqueous ClO_2_ treatment (80 ppm for 15 min) yielded an approximately 1.5–3 log reduction in aerobic bacteria in mulberry (Chen et al., 2011). Similarly, a 10 min exposure to 100 ppm ClO_2_ significantly decreased the initial populations of natural microflora in blueberries (Chun et al., 2013). With gaseous ClO_2_ application (5.5 ppm), >5 log reduction in the load of artificially inoculated *Salmonella* spp., was observed in whole blueberries and strawberries (Annous et al., 2020). Similar results were observed for *L. monocytogenes*, *E. coli* O157:H7, yeast, and molds (Mahmoud et al., 2007; Popa et al., 2007; Wu and Kim, 2007). However, ClO_2_ efficacy was higher for *Salmonella* inoculated on blueberry skin tissues than for those inoculated on stem scar tissues (Sy et al., 2005a). Sun et al. (2014) reported an approximately 4 log reduction in the load of *Colletotrichum acutatum* on blueberries with ClO_2_ fumigation. Berries treated with ClO_2_, generated in a small chamber with acidified sodium chlorite solution, reduced Tulane virus populations by >1–3.3 log (Kingsley et al., 2018; Kingsley and Annous, 2019).

Previous studies have investigated the effects of various concentrations and exposure times of ClO_2_ on the populations of *E. coli* O157:H7 and *L. monocytogenes* on the skin surface, stem, and calyx cavities of apples. Although an exposure of 4.0 ppm ClO_2_ for 10 min resulted in a 5.5 log reduction in *L. monocytogenes* populations, treatment with 12.0 ppm ClO_2_ for 10 min, 7.2 ppm ClO_2_ for 20 min, or 4.8 ppm ClO_2_ for 30 min completely suppressed the bacterial population, which was initially inoculated on the skin (Du et al., 2002, 2003). Moreover, after 3 h of exposure to low ClO_2_-releasing sachets, the population of *Alicyclobacillus acidoterrestris* spores decreased to 4.5 log on apple surface (Lee et al., 2006). A 10 min-long fumigation with 0.5 ppm ClO_2_ on oranges resulted in >5 log reduction in *Salmonella* load (Bhagat et al., 2011). Gaseous ClO_2_, at concentrations 200−1,800 ppm, significantly lowered the incidence of green mold on citrus fruits, including kumquats, mandarins, and Peru oranges, and *Penicillium digitatum* on grapefruits (Liu et al., 2020). Furthermore, ClO_2_ treatment effectively reduced *Xanthomonas citri* contamination in both artificially and naturally contaminated citrus fruits (Behlau et al., 2021). Reports suggest that ClO_2_ treatment is more effective against *E. coli* inoculated on smooth non-stem-scar surfaces than on rough stem-scar areas (Pao and Davis, 1999). Additionally, *X. citri* on grapefruit surface requires a higher ClO_2_ concentration for complete inactivation than *E. coli* (Sun et al., 2017a).

### Long-Term Effects

Postharvest storage is essential for some types of fresh produce; however, microbial populations gradually increase during their storage. Hence, a strong disinfection method is required to ensure long-term protection of the treated produce. An initial reduction of microbial load is important for extending the microbiological shelf-life of fresh produce (Lin et al., 2021). Previous studies have reported different efficacies of ClO_2_ in inhibiting microbial growth during postharvest storage of produce (Praeger et al., 2018; Singh et al., 2021). A 20 min exposure to aqueous ClO_2_ inhibited the growth of natural microflora and prolonged the shelf-life of asparagus lettuce for 10 days (Chen et al., 2010). In ClO_2_-treated fresh produce, including apples, green leaf lettuce, cantaloupe, and strawberries, the populations of inoculated pathogens remained relatively unchanged, whereas the growth of natural microflora was significantly delayed after 9 days of storage at 4°C (Rodgers et al., 2004). In lettuce, ClO_2_ treatment (5.0 ppm for 10 min) maintained the populations of mesophilic and psychrotrophic bacteria, yeast, and mold under the detectable limit for 5 days at 4°C (Mahmoud and Linton, 2008). Similarly, ClO_2_-treated tomatoes exhibited significantly low microflora abundance during a storage period of 28 days; however, the efficacy of ClO_2_ varied with the exposure time and ClO_2_ concentration (Trinetta et al., 2010). Bhagat et al. (2010) demonstrated that treating tomato surface with 0.5 ppm ClO_2_ gas for 12 min delayed the growth of natural microflora and extended its shelf-life by 7 days during storage at 22°C. Furthermore, ClO_2_ treatment significantly delayed the development of white molds and black spots in Roma tomato wounds inoculated with *S. vesicarium* and *A. alternate* (Trinetta et al., 2013). Controlled release of ClO_2_ (4–6 ppm) reduced the loads of *E. coli*, *Salmonella*, and *A. alternata* on tomatoes by 3–5 log by the end of a 14 days-storage period (Sun et al., 2017b). Moreover, strawberries packed with ClO_2_-generating pads exhibited reduced growth of yeast and molds until 8 days of their 12 days-storage period at 2°C (Chiabrando et al., 2018). Similarly, ClO_2_ treatment reduced total aerobic bacterial and yeast and mold counts by 0.95 and 0.94 log, respectively, in grape fruit after 6 weeks of storage at 10°C (Sun et al., 2017a).

By contrast, ClO_2_ exhibits no long-term effects on reducing microbial contamination despite its initial effect. Treating cucumbers with various concentrations of ClO_2_, ranging from 20 to 125 ppm, did not delay mold growth during storage (Praeger et al., 2018). Although ClO_2_ treatment, in combination with MAP, was effective in controlling microflora on mungbean sprouts during storage, ClO_2_ treatment alone could not reduce the incidences of *S. typhimurium* and *L. monocytogenes* (Jin and Lee, 2007). ClO_2_ treatment of fresh-cut lettuce packed in MAP did not inhibit the growth of yeast during storage (López-Gálvez et al., 2010). However, 3 and 5 ppm ClO_2_ were more effective against *L. monocytogenes* than yeasts and molds during cold storage (Rodgers et al., 2004).

## Effects of Chlorine Dioxide Treatment on the Postharvest Quality of Fresh Produce

### Color

Color is one of the fundamental characteristics that determines the visual quality and acceptability of fresh produce. Depending on the concentration, ClO_2_ differentially affects the appearance of treated fresh produce (Table 4). However, previous studies suggest that ClO_2_ has no effect on the color of fresh produce; ClO_2_ exposure had no effect on Hunter L, a, and b values of tomatoes, spinach, and lettuce (Kim et al., 2007; Song et al., 2011; Hassenberg et al., 2014; Park and Kang, 2015a). Similarly, treatment with 0.5 ppm ClO_2_ gas for 12 min did not significantly affect the color of orange peel (Bhagat et al., 2011). Furthermore, the appearance of blueberries was not affected by long-term ClO_2_ exposure (2–12 h) (Popa et al., 2007; Wu and Kim, 2007). By contrast, higher concentrations of ClO_2_ result in the bleaching of fresh produce. For example, strawberries treated with ClO_2_ underwent white bleaching after 8 days of storage at 2°C (Chiabrando et al., 2018). Oxidation of oligosaccharides, such as cellulose and hemicellulose, and chlorophyll, has been hypothesized as the possible cause of bleaching in fresh produce (Singh et al., 2002; Chen and Zhu, 2011).

**TABLE 4:** Effects of chlorine dioxide on color and visual quality of fresh produce.

Produce	Mode	ClO_2_ concentration	Duration	Storage	ClO_2_ effect	References
**Color**						
Lettuce	Aq	0, 5, 10, 20 ppm	10 min	4 days, 4°C	Unaffected	Kim et al., 2008
Lettuce	Aq	50 ppm	10 min	8 days, 4°C	Unaffected	Kim et al., 2007
Lettuce	Aq	10, 40, 100 ppm	5, 10, 20 min	14 days, 4°C	Delayed degradation of color	Chen et al., 2010
Lettuce	G	0.5, 5.0 ppm	2, 10 min	7 days, 4°C	Leaf discoloration	Mahmoud and Linton, 2008
Lettuce	G	1.4 ppm	5.4–10.5 min	10 days, 10°C	Slight leaf browning	Sy et al., 2005b
		2.7 ppm	10.4–20.0 min		Leaf browning	
		4.1 ppm	20.5–30.8 min		Neaf browning	
Spinach	G	1–30 ppm	20 min	7 days, 4°C	Unaffected	Park and Kang, 2015b
		50 ppm			Hiher L* and b* values	
Cabbage	G	1.4 ppm	5.4–10.5 min	10 days, 10°C	Slight browning	Sy et al., 2005b
		2.7 ppm	10.4–20.0 min		Leaf browning	
		4.1 ppm	20.5–30.8 min		Leaf browning	
Tomato	Aq	10 ppm	5 min	10 days, 4°C	Unaffected	Song et al., 2011
		50 ppm	20 min		Discoloration	
Tomato	G	8 ppm	60 s	25°C, 28 days	Unaffected	Trinetta et al., 2010
Tomato		10 ppm	120, 180 s	25°C, 28 days	Skin wrinkling	Trinetta et al., 2010
Tomato	G	0.5 ppm	12 min	28 days, 22°C	Unaffected	Bhagat et al., 2010
Tomato	G	1.4 ppm	6 min	10 days, 21°C	Unaffected	Sy et al., 2005b
		2.7 ppm	12 min		Unaffected	
		4.1 ppm	25 min		Unaffected	
Carrot	G	1.4 ppm	5.4–10.5 min	10 days, 10°C	Slight whitening in the color	Sy et al., 2005b
		2.7 ppm	10.4–20.0 min		Whitening in the color	
		4.1 ppm	20.5–30.8 min		Whitening in the color	
Onions	G	1.4 ppm	5.4 min	12 or 20 days, 21°C	Unaffected	Sy et al., 2005b
		2.7 ppm	10.4 min		Unaffected	
		4.1 ppm	20 min	.	Unaffected	
Apple	G	1.4 ppm	6 min	41 days, 21°C	Unaffected	
		2.7 ppm	12 min		Unaffected	
		4.1 ppm	25 min		Small brown spots on the skin	
Cantaloupe	G	0.5–5.0 mg/L	0–10 min	12 days, 22°C	Unaffected	Mahmoud et al., 2008
Strawberry	G	0.5–5 ppm	10 min	16 days, 4°C	Unaffected	Mahmoud et al., 2007
		29 ppm			Peel browning	
Strawberry	G	NA	NA	3 days, 4°C + 2 days at 20°C/12 days, 2°C	Unaffected	Chiabrando et al., 2018
				12 days, 2°C	Unaffected	
Strawberry	Aq	5 ppm	NA	3 weeks, 4°C	Maintained L and a values	Aday and Caner, 2011
					Decreased L and a values	
Peaches	G	1.4 ppm	5.4 min	10 days, 21°C	Browning	Sy et al., 2005b
		2.7 ppm	10.4 min	.	Browning	
		4.1 ppm	20 min	.	Browning	
**Visual quality**						
Tomato	G	5 ppm	12 h	20 days, 5°C	Delayed color development	Islam et al., 2017
Apple	G	0.39–0.60 ppm	1–3 h	7 days, 4°C	Unaffected	Lee et al., 2006
		1.78–2.69 ppm	1–3 h		Black spots on the fruit surface	
		4.32–6.55 ppm	1–3 h		Black spots on the fruit surface	
Grapefruit	G	14.5 ppm	10 days	42 days, 10°C + 7 days, 20°C	Maintained	Sun et al., 2017a
		29 ppm			Peel browning	

*Aq, aqueous; G, gaseous; NA, not available.*

Chlorine dioxide has been reported to differentially affect enzymatic browning of fresh produce, resulting from the oxidation of phenols to o-quinones that is catalyzed by polyphenol oxidase (PPO; Altunkaya and Gökmen, 2009) during postharvest handling and processing. In grapes, repeated application of ClO_2_ during storage significantly decreased rachis browning (Chen et al., 2018). Reduced browning in a variety of fresh produce, such as fresh-cut asparagus lettuce, and apples, is associated with decreased PPO activity (Fu et al., 2007; Chen et al., 2010). This can be attributed to the oxidation of disulfide bonds and amino acids at the active site of PPO by ClO_2_ (Fu et al., 2007). By contrast, ClO_2_ treatment may also cause browning of fresh produce. For example, ClO_2_ treatment resulted in rapid color change in spinach leaves, browning of grapefruit, cabbage, lettuce, peaches, and apples (Sy et al., 2005b; Lee et al., 2006; Mahmoud and Linton, 2008; Park and Kang, 2015a; Sun et al., 2017a).

### Firmness

Firmness, another important quality-determining characteristic, influences consumer appeal and the commercial value of fresh produce. Effect of ClO_2_ treatment on firmness and weight loss of fresh produce is summarized in Table 5. ClO_2_ treatment retains the firmness of several fresh fruits, such as strawberries, plums, apricots, and mangoes, during postharvest storage (Aday and Caner, 2011; Chen and Zhu, 2011; Zhang et al., 2019). Furthermore, controlled-release of ClO_2_ gas has been reported to regulate the firmness of non-inoculated and *E. coli*- and *C. acutatum*-inoculated berries during storage (Sun et al., 2014).

**TABLE 5:** Effects of chlorine dioxide on firmness and weight loss of fresh produce.

Produce	Mode	ClO_2_ concentration	Duration	Weight loss	Firmness	Storage	References
Spinach	G	1–30 ppm	20 min		Unaffected	7 days, 4°C	Park and Kang, 2015b
Spinach	G	1–50 ppm	20 min		Unaffected	7 days, 4°C	Park and Kang, 2015b
Grape tomatoes	G	2–3.5 ppm	14 days	Reduced	Increased	14 days, 20°C	Sun et al., 2017b
Grape tomatoes	G	2, 4, 6, 8 ppm	14 days	Reduced	Increased	14 days, 20°C	Sun et al., 2017c
Cherry tomatoes	G	2, 4, 6, 8 ppm	14 days	Reduced	Maintained	14 days, 20°C	Sun et al., 2017c
Tomato	G	10 ppm	120, 180 s		Skin wrinkling	25°C, 28 days	Trinetta et al., 2010
Tomato	G	5 ppm	12 h	Reduced	Increased	20 days, 5°C	Islam et al., 2017
Strawberry	Aq	5 ppm	NA		Reduced	3 weeks, 4°C	Aday and Caner, 2011
		10 ppm			Increased		
Blueberry	G	1–2.5 ppm	9 days		Maintained	9 days, 10°C	Sun et al., 2014

*Aq, aqueous; G, gaseous; NA, not available.*

After harvesting, respiration and transpiration continue in fresh produce, and carbohydrate and water reserves are continually consumed without replacement, leading to progressive loss of turgidity and weight during storage. Fruit moisture and weight loss are associated with decreased fruit firmness, shrinking, and shriveling (Paniagua et al., 2013; Lufu et al., 2020). However, ClO_2_ reduces the rate of water loss in the ClO_2_-treated produce (Guo et al., 2014; Wang et al., 2014). The application of ClO_2_ at low concentrations for long durations in active packaging material has been shown to improve fruit firmness and reduce water loss (Guo et al., 2014).

Less weight loss in ClO_2_-treated berries is associated with 50% closed stomata during storage at low temperatures (Wang et al., 2014). In general, fruit ripening is associated with a climacteric increase in ethylene production and extensive modifications in cell wall polysaccharides. ClO_2_ delays the increase in respiration rate and ethylene biosynthesis, resulting in delayed ripening that further leads to delayed fruit softening (Chen and Zhu, 2011; Guo et al., 2013, 2014). ClO_2_ may also alter tissue metabolism by oxidizing cell constituents, thereby leading to changes in respiration, and, in turn, inhibiting weight loss and maintaining fruit firmness (Gomez-Lopez et al., 2008).

### Sensory Properties

Previous studies have reported that ClO_2_ treatment can retain the sensory properties of fresh produce. Effect of ClO_2_ treatment on sensory properties of fresh produce is summarized in Table 6. For example, gaseous ClO_2_ treatment (4.1 ppm) did not compromise the sensory qualities of blueberries, strawberries, and raspberries stored for 10 days at 8°C (Sy et al., 2005a). Similar results were obtained for fresh-cut cabbage, carrot, and iceberg lettuce treated with 3–5 ppm ClO_2_ (Rodgers et al., 2004; Sy et al., 2005b; López-Gálvez et al., 2010). Moreover, ClO_2_ treatment positively affects the composition of volatile compounds and free amino acids in citrus fruits, resulting in the retention of their distinct flavor (Liu et al., 2020). Furthermore, ClO_2_-treated plums maintain high sensory properties during storage (Chen and Zhu, 2011). Few studies revealed that the sensory properties of fresh produce can be improved by ClO_2_ application. For instance, ClO_2_-treated strawberries, blueberries, and mulberries exhibited better sensory scores than the untreated controls (Jin and Lee, 2007; Wu and Kim, 2007; Chen et al., 2011; Chun et al., 2013). Nonetheless, 20 ppm ClO_2_ significantly affected the sensory properties of lettuce and cabbage (Gomez-Lopez et al., 2008).

**TABLE 6:** Effects of chlorine dioxide on sensory properties of fresh produce.

Produce	Mode	ClO_2_ concentration	Duration	Storage	Sensory property	References
Lettuce	Aq	3 ppm	1 min	3 days, 4°C + 7 days, 8°C	Unaffected	López-Gálvez et al., 2010
Lettuce	Aq	50 ppm	10 min	8 days, 4°C	Unaffected	Kim et al., 2007
Lettuce	Aq	3, 5 ppm		48 h, 4°C	Unaffected	Rodgers et al., 2004
Lettuce	Aq	10, 40, 100 ppm	5, 10, 20 min	14 days, 4°C	Unaffected	Chen et al., 2010
Lettuce	G	1.4 ppm	5.4–10.5 min	10 days, 10°C	Decreased	Sy et al., 2005b
Cabbage	G	1.4 ppm	5.4–10.5 min	10 days, 10°C	Decreased	Sy et al., 2005b
Carrot	G	1.4 ppm	5.4–10.5 min	10 days, 10°C	Decreased	Sy et al., 2005b
Tomato	G	1.4 ppm	6 min	10 days, 21°C	Unaffected	Sy et al., 2005b
Onions	G	1.4 ppm	5.4 min	12 or 20 days, 21°C	Unaffected	Sy et al., 2005b
Apple	Aq	3, 5 ppm		48 h, 4°C	Unaffected	Rodgers et al., 2004
Strawberry	Aq	3, 5 ppm		48 h, 4°C	Unaffected	Rodgers et al., 2004
Strawberry	Aq	5 ppm		3 weeks, 4°C	Maintained	Aday and Caner, 2011
					Maintained	
Cantaloupe	Aq	3, 5 ppm		48 h, 4°C	Unaffected	Rodgers et al., 2004
Blueberry	G	4 ppm	12 h	Overnight, 4°C	Improved	Popa et al., 2007
Apple	G	1.4 ppm	6 min		Unaffected	
Peaches	G	1.4 ppm	5.4 min	10 days, 21°C	Decreased	Sy et al., 2005b
		2.7 ppm	10.4 min		Decreased	
		4.1 ppm	20 min		Decreased	
Grapefruit	G	14.5 ppm	10 days	42 days, 10°C + 7 days, 20°C	Maintained	Sun et al., 2017a

*Aq, aqueous; G, gaseous.*

## Chlorine Dioxide Application: Efficacy and Limitations

Because of its high oxidative capacity (2.5-fold that of chlorine), ClO_2_ is effective in microbial inactivation at concentration as low as 0.1 ppm with minimal contact time (Praeger et al., 2018). Most importantly, ClO_2_ is effective against both Gram-positive and Gram-negative bacteria, whereas molds and yeasts showed intermediate tolerance (Yoon and Lee, 2018; Sun et al., 2019). Additionally, ClO_2_ does not react with organic matter to form carcinogenic by-products such as trihalomethanes which makes ClO_2_ to be effective over a wide pH range (Praeger et al., 2018). In the United States, a maximum 3 ppm of ClO_2_ is allowed for fresh produce treatment. In Europe, rinsing with potable water is necessary following the ClO_2_ treatment (Praeger et al., 2018).

Comparison of disinfection efficacy of various sanitizers revealed that gaseous ClO_2_, hydrostatic pressure and electrolyzed oxidizing water were more effective in microbial inactivation than other sanitizers. The average microbial reductions of ClO_2_ gas, hydrostatic pressure and electrolyzed oxidizing water were 4.07, 3.94, and 3.01 log, respectively (Yoon and Lee, 2018). On the other hand, the average microbial inactivation of aqueous ClO_2_ (1.49 log) was less than gaseous ClO_2_, however, it was still higher than chlorine-based disinfectants (1.12 log) (Yoon and Lee, 2018). Higher antimicrobial activity of gaseous ClO_2_ may attribute to its easier accessibility to microbes located in the unreachable parts the fresh produce. Moreover, ClO_2_ gas can readily diffuse into the tissues of fresh produce, hence, it may inactivate internalized microbes (Yoon and Lee, 2018). However, handling with gaseous ClO_2_ is inconvenient as it needs to be produced onsite. Moreover, it is expensive and requires technical expertise.

The major limitations of ClO_2_ for practical applications include it may not be effective at permitted concentrations; it may affect quality of treated fresh produce in some instances. Since ClO_2_ is highly explosive and toxic to humans at higher concentrations, it is challenging to implement this treatment technology at industry scale.

## Conclusion and Future Perspectives

Chlorine dioxide application, in gaseous and aqueous forms, has been demonstrated to be effective in controlling microbial growth and retaining the quality of fresh produce, however, it is largely depending upon the respective produce type and treatment conditions. Gaseous ClO_2_ is more effective than the aqueous form. Nevertheless, although aqueous ClO_2_ solutions may be easy to use, they require an additional washing step. ClO_2_, whether in gaseous or aqueous form, destabilizes cell membranes, alters membrane permeability, and interrupts protein synthesis in microbes, along with influencing ethylene biosynthesis and respiration rate in fresh produce, which are crucial for maintaining the quality of fresh produce. In general, initial reduction in microbial load significantly affects microbial contamination during storage of fresh produce, thereby resulting in an extended shelf-life. Previous studies suggest that ClO_2_ concentration and exposure time are crucial in determining the efficacy of ClO_2_ against microbes, but a holistic approach is required to unravel the mechanisms underlying the regulation of fresh produce quality by ClO_2_.

Our review showed that current research on disinfection by ClO_2_ has mainly focused on the bactericidal effects of ClO_2_; recently, studies on antifungal and antiviral effects of ClO_2_, are gaining attention. Currently, the efficacy of ClO_2_ has been mostly tested at the laboratory level, thus, highlighting the need for industrial-level testing for various types of fresh produce. Disposition and chemical fate of ClO_2_ gas on treated fresh produce are not well understood; therefore, further studies should focus on this dimension, which has been largely neglected in studies on ClO_2_ disinfection. Moreover, this review did not assess the different methods of ClO_2_ generation and the efficacy of ClO_2_ in combination with other technologies for postharvest quality and microbial safety of fresh produce.

## Author Contributions

M-HP: supervision. SM and M-HP: conceptualization, writing–original draft preparation, contributed to the article, and approved the submitted version.

## Conflict of Interest

The authors declare that the research was conducted in the absence of any commercial or financial relationships that could be construed as a potential conflict of interest.

## Publisher’s Note

All claims expressed in this article are solely those of the authors and do not necessarily represent those of their affiliated organizations, or those of the publisher, the editors and the reviewers. Any product that may be evaluated in this article, or claim that may be made by its manufacturer, is not guaranteed or endorsed by the publisher.

## Abbreviations

MAP: modified atmosphere packaging
PPO: polyphenol oxidase
PPM: parts per million.
